# Cerebral venous thrombosis after COVID-19 vaccination: is the risk of thrombosis increased by intravascular application of the vaccine?

**DOI:** 10.1007/s15010-021-01658-x

**Published:** 2021-07-21

**Authors:** Lutz Gürtler, Rainer Seitz, Wolfgang Schramm

**Affiliations:** 1 Ludwig-Maximilians University (LMU), Max von Pettenkofer Institute, Munich, Germany; 2 Present Address: Sofienstraße 70, 63225 Langen, Germany; 3 Ludwig-Maximilians University (LMU) and Rudolf Marx Stiftung, Munich, Germany

**Keywords:** Venous thrombosis, SARS-CoV-2 vaccination, Adenovirus vector, PF4 antibody, Thrombocytopenia

Currently, an unprecedented campaign of vaccination against COVID-19 is carried out worldwide, involving many millions of people. Pre-licensing clinical studies of new vaccines can include only a limited number of subjects; therefore rigorous pharmacovigilance is warranted to detect rare side effects.

The surveillance of the recombinant adenovirus-based COVID-19 vaccine ChAdOx1-S (Oxford, Astra-Zeneca) which contains chimpanzee adenovirus (AdV) encoding the SARS-CoV-2 spike glycoprotein, revealed rare thromboses with concurrent thrombocytopenia, including venous thromboses in unusual sites such as cerebral venous sinus thrombosis (CVST), intestinal venous and arterial thromboses [1]. Soon thereafter, the Pharmacovigilance Risk Assessment Committee (PRAC) of the European Medicines Agency (EMA) reported also a signal concerning thromboembolic events with thrombocytopenia after vaccination with the Ad26.COV2-S COVID-19 vaccine (Johnson & Jonhnson) [2], which contains the cloned SARS-CoV-2 spike glycoprotein in AdV type 26. Both above-mentioned vaccines are abbreviated in the following as AdV-S. The above described thrombotic complications after AdV-S have in the meantime be named TTS (thrombosis thrombocytopenia syndrome), VIPIT (vaccine-induced prothrombotic immune thrombocytopenia) or VITT (vaccine-induced thrombocytopenia) [3–5].

For other SARS-CoV-2 vaccines, which do not use adenovirus vectors, similar observations were only reported until now in two women [6, 7].

Most of the cases reported to date have occurred in persons under 60 years of age within 2 weeks of receiving the first vaccine dose [3]. Since females consented more frequently to be vaccinated there were more female than male vaccinees involved, but this observation is in line with the experience concerning risk factors for cerebral venous sinus thrombosis [8–11]. The occurrence of thrombosis focused on CVST after AdV-S vaccination, and has to be balanced with the frequency of spontaneous cerebral venous sinus thrombosis [12], and CVST associated with SARS-CoV-2 infection [13, 14]. The time period of the occurrence of CVST in COVID-19 patients even under anti-thrombotic treatment is reported to be ca 3–30 days after onset of infection [3, 15]. It was reported that in patients with COVID-19 pneumonia an incidence between 7.7 and 28% of cerebral thrombosis was found in Intensive Care Units, dependent on age, gender, comorbidities, and cytokine release, compared to a general incidence of 2–5–15 cases per million people [9]. The frequency of atypical thrombosis after vaccination has to be weighted with that of thrombosis in the general population [16], and the higher frequency of thrombosis in COVID-19 patients in emergency departments, which will be strongly reduced by vaccination [17, 18]. Factors contributing to the preferential affection of female gender in the observed thromboses after AdV-S vaccination may include increased seasonal adenovirus production supported by beta-estradiol [19] and hormonal activation of thrombocytes [20]. There may be further some contribution to a tendency of hypercoagulation by genetic factors, since the kinetics of clot formation after vaccination is at least fivefold enhanced when there are mutations in von Willebrand factor cleaving protease deficiency G20210A (ADAMTS-13) [10], and presence of factor V Leiden mutation, enhanced by oral contraceptive use [21–23], which however were not an apparent cause of most of the so far observed cases of atypical thrombosis after vaccination [24, 25].

Findings in several of the first patients with unusual thrombosis after AdV-S vaccination showed similarities with heparin-induced thrombocytopenia (HIT) with high levels of antibodies to platelet factor 4 (PF4)–polyanion complexes [5, 24–26]. PF4 is synonymous with CXCL4, a potent cytokine which is released from activated thrombocytes and associated with vaccine-induced immune thrombotic thrombocytopenia [27, 28]. Patient sera activated platelets [24, 25] more strongly in the presence of platelet factor 4; blocking experiments demonstrated that platelet activation had occurred through platelet Fcγ receptors [24]. The authors suggested that it remains to be elucidated whether the antibodies found are autoantibodies against PF4 induced by the strong inflammatory stimulus of vaccination or antibodies induced by the vaccine that cross-react with PF4 and platelets [24]. In the context of the platelet-activating antibodies against PF4 found after vaccination with AdV-S vaccine, it may be interesting that adenovirus binds to negatively charged glycosaminoglycans like heparan sulfate [29]. The concept of the pathogenesis of HIT [30] includes that serial PF4 molecules become aligned by binding to heparin, by binding anti-PF4 IgG molecules, which leads to the formation of large complexes. The antibodies involved in thrombocytopenia after AdV-S vaccination occurred without any prior heparin therapy, and their effect on platelet activation was rather blocked by heparin [24]. One might speculate that the presumably positively charged structures on the AdV surface which bind negatively charged glycosaminoglycans, might elicit antibodies cross-reacting with PF4, which would explain the finding that both PF4 and the AdV components of the vaccine enhanced platelet activation [5].

AdV have been widely used as vectors for gene therapy. Intravascular delivery of AdV-S elicits both a primary innate immunological response to the vector, characterized by rapid increases in levels of serum cytokines and chemokines, and subsequent adaptive immunological responses that restrict repeated administration of the vector. Moreover, intravascular application of AdV-based vaccines is limited by the inherent hepatic tropism of some AdV and by the associated host inflammatory responses to the vector [31, 32], as well as interaction with platelets [33], endothelial cells, and coagulation cascade [26, 34]. Thus, AdV-S vaccine invading the bloodstream may directly contribute to thrombocyte activation [5]. PEGylating of AdV5 used as a vector for gene therapy reduced the toxicity, clotting and complement activation, cytokine release in human blood, while iv application of the AdV-5 might be associated with cytokine storm, hypercoagulation and liver cell necrosis [35]. Factor X (FX) binds to the hexon of AdV5 and promotes entry to hepatocytes, and activation of the TRL4 related pathway [34].

To reduce side effects the present AdV-S vaccine is based on AdV encoding the S1 glycoprotein of SARS-CoV-2 corresponding to 2.5 × 10^8^ infectious units (IU) (manufacturer’s information). The vaccine has to be injected IMintramuscularly and the cloned AdV will attach to myocytes, fibroblasts, dendritic cells, macrophages and further cells near the injection site as antigen-presenting cells [29]. The cloned virus induces a strong immune response [36] especially after the second boost injection of the vaccine with a broad T-cell immune response and production of IgG antibodies [37] that are distributed by the lymphogenic and haematogenic route. In blood AdV will attach to all cells, including erythrocytes [38] and platelets, heparan, heparan sulfate, glycosaminoglycan [29], and activate platelets [34], form complexes, that might attach to endothelial cells and induce cytokine release [5, 31]. A further way of complex formation is the liberation of AdV-DNA by antigen-presenting cells, by microtrauma or microbleeding resulting in intravascular access and attachment of AdV-DNA to PF4 and finally leading to VITT [5]. DNA shares electrochemical similarities with heparin, binds to PF4, and complexes with PF4 autoantibodies which are found more frequently in younger females [5]. When these complexes circulate in the bloodstream they might be dispersed during their passage through capillaries or might grow in sites prone for turbulences and shearing forces as for example in the carotid sinus [39] attach to the endothelium and finally cause CVST [14, 25].

According to current recommendations [40], aspiration before IM intramuscular injection of vaccines or toxoids (i.e., pulling back on the syringe plunger after IM intramuscular needle insertion, but before injection) is no longer necessary because no large blood vessels are present at the recommended deltoid muscle injection site. If by chance the vaccine is injected into a blood vessel, the AdV-S will attach to blood cells and endothelial cells, and activate thrombocytes. After 1–2 weeks activated thrombocytes might lead to the formation of complexes of blood cells, in the presence of PF4 antibodies and components of the clotting system and possibly lead to thrombosis [3, 5, 25]. Regarding the kinetics and time and frequency of thrombosis formation after vaccination, there is room for speculation that clot formation would be fostered by intravascular application of the vaccine, which still is a rare event of < 1 in 100,000 [5]. Whether a technical reason- no aspiration, injection at the wrong site-, or anatomical reason aberrant large vessels, presence of haemangioma-, or immunological reason e.g. hyperreactivity of thrombocytes contribute to the pathological reaction to AdV-S vaccine application gives room for further investigation in vaccine application, especially on the technical site.

The overall risk–benefit balance in favour of vaccination remains clearly positive [37]. However, according to the bad experiences with the initial intravascular AdV vector-based gene therapy, adverse effects by the intravascular application of AdV-S vaccine should be avoided, and a strictly IM intramuscular injection should be warranted.
